# Puerarin mitigated LPS‐ATP or HG‐primed endothelial cells damage and diabetes‐associated cardiovascular disease via ROS‐NLRP3 signalling

**DOI:** 10.1111/jcmm.18239

**Published:** 2024-05-22

**Authors:** Huizhen Wei, Mengru Sun, Ruixuan Wang, Hairong Zeng, Bei Zhao, Shenyi Jin

**Affiliations:** ^1^ Shanghai Frontiers Science Center of TCM Chemical Biology, Institute of Interdisciplinary Integrative Medicine Research Shanghai University of Traditional Chinese Medicine Shanghai China; ^2^ Department of Endocrinology Shuguang Hospital Affiliated to Shanghai University of Traditional Chinese Medicine Shanghai University of Traditional Chinese Medicine Shanghai China

**Keywords:** diabetic vasculopathy, Puerarin, pyroptosis, ROS

## Abstract

The occurrence and development of diabetic vascular diseases are closely linked to inflammation‐induced endothelial dysfunction. Puerarin (Pue), the primary component of *Pueraria lobata*, possesses potent anti‐inflammatory properties. However, its vasoprotective role remains elusive. Therefore, we investigated whether Pue can effectively protect against vascular damage induced by diabetes. In the study, Pue ameliorated lipopolysaccharide‐adenosine triphosphate (LPS‐ATP) or HG‐primed cytotoxicity and apoptosis, while inhibited reactive oxygen species (ROS)‐mediated NLR family pyrin domain containing 3 (NLRP3) inflammasome in HUVECs, as evidenced by significantly decreased ROS level, NOX4, Caspase‐1 activity and expression of NLRP3, GSDMD, cleaved caspase‐1, IL‐1β and IL‐18. Meanwhile, ROS inducer CoCI_2_ efficiently weakened the effects of Pue against LPS‐ATP‐primed pyroptosis. In addition, NLRP3 knockdown notably enhanced Pue's ability to suppress pyroptosis in LPS‐ATP‐primed HUVECs, whereas overexpression of NLRP3 reversed the inhibitory effects of Pue. Furthermore, Pue inhibited the expression of ROS and NLRP3 inflammasome‐associated proteins on the aorta in type 2 diabetes mellitus rats. Our findings indicated that Pue might ameliorate LPS‐ATP or HG‐primed damage in HUVECs by inactivating the ROS‐NLRP3 signalling pathway.

## 1 INTRODUCTION

Diabetes mellitus (DM), a chronic metabolic disorder, is marked by either an absolute or relative deficiency in insulin, prolonged hyperglycaemia and insulin resistance. This figure is expected to surge further as the incidence rate continues to climb. Prolonged exposure to elevated blood glucose levels can trigger chronic microangiopathy, affecting various organs such as the cardiovascular system, kidneys, retina and nervous system, posing significant risks to human life and health.^1^, ^2^ While the pathogenesis of cardiovascular complications linked to diabetes is complex, endothelial dysfunction is widely acknowledged as a key factor in the development of vascular issues in type 2 diabetes mellitus (T2DM).^3^

Endothelial dysfunction is a primary step in the pathogenesis of vascular complications in T2DM, hallmarked by elevated inflammation.^4^ Under normal conditions, endothelial cells remain quiescent and modulate vascular tension. In the progression of T2DM, hyperglycaemia will cause endothelial cell dysfunction. Diabetic vascular complications are the leading causes of death, usually induced by inflammation‐induced endothelial cell impairment.^5^, ^6^ The factors that contribute to endothelial dysfunction in diabetes include inflammation, hyperglycaemia and oxidative stress.^7^, ^8^ Recent studies demonstrated that suppression of inflammation may protect T2DM rats from an aortic impairment, indicating the potential of vasoprotection in diabetes by targeting inflammation.

Pyroptosis is a unique form of inflammatory cell death hallmarked by plasma membrane rupture and subsequent release of cellular contents and pro‐inflammatory mediators, which involves two key molecular events, that is, activation of inflammasomes and caspase‐1.^9^ The relationship between pyroptosis and apoptosis involves cross‐regulation, where evidence suggests that inhibiting or promoting one of these death pathways under specific conditions may impact the occurrence of the other. Additionally, shared pathways are evident through proteins like caspase‐3 and caspase‐8, which play dual roles in both the execution phase of apoptosis and the regulation of pyroptosis, emphasizing their crucial functions within the interconnected mechanisms of these two cellular death processes. Pyroptosis intricately contributes to the pathogenesis of diabetic vascular damage and serves as a key factor exacerbating vascular complications in the context of diabetes. This implication extends to diabetic vasculopathy, where the aberrant activation of the NLR family pyrin domain containing 3 (NLRP3) inflammasome and subsequent pyroptotic cascade notably play a role in the development of DCM. Furthermore, the association between pyroptosis and diabetic retinopathy (DR) is evident, with pyroptosis critically contributing to the adverse effects experienced by cells in the retinal neurovascular unit (NVU) under hyperglycaemic conditions. Moreover,^10^, ^11^, ^12^, ^13^, ^14^ the activation of the nucleotide oligomerization domain (NOD)‐like receptor (NLR) family, pyrin domain‐containing protein 3 (NLRP3) inflammasomes are associated with the pathogenesis of a wide range of inflammatory diseases. The activation of NLRP3 can facilitate caspase‐1‐mediated proteolytic activation of IL‐1β and IL‐18. Mounting evidence has indicated that NLRP3 inflammasome activation is vital for the development of diabetes‐related atherosclerosis.^15^, ^16^ Thus, repression of the NLRP3 inflammasome signalling pathway contributes to alleviate diabetes‐related atherosclerosis.^17^, ^18^ Recent studies have found that reactive oxygen species (ROS) can cause a variety of inflammatory responses by activating NLRP3 inflammasome under pathological conditions.^19^ It has been reported that suppression of ROS‐NLRP3 inflammasomes contributed to protect endothelial cells from pyroptosis.

Traditional Chinese medicine is a promising treatment for diabetic vasculopathy. Puerarin (Pue) (7,4′‐dihydroxyisoflavone‐8‐β‐glucopyranoside) derived from the root of *Pueraria lobata* is the main bioactive ingredient used for the treatment.^20^, ^21^, ^22^, ^23^ Pue has the effect of treating hyperglycaemia‐related diseases containing diabetic nephropathy, diabetic macroangiopathy, diabetic retinopathy, diabetic cardiomyopathy and diabetic peripheral neuropathy.^24^, ^25^, ^26^ Notably, previous studies reported that Pue exerted antioxidant and anti‐inflammatory effects, especially for the inhibition of ROS and NLRP3 inflammasome.^27^, ^28^ The specificity of Pue in addressing diabetes‐associated cardiovascular disease requires further clarification. This inquiry seeks to establish a coherent understanding of the mechanisms by which Pue exerts its effects on oxidative stress, inflammasome activation and pyroptotic cell death specifically within the context of diabetic vascular complications.

In this study, we employed lipopolysaccharide‐adenosine triphosphate (LPS‐ATP) or high glucose (HG)‐primed human umbilical vein endothelial cells (HUVECs) as a cellular model and established a high‐fat diet (HFD) and streptozotocin (STZ)‐induced type 2 diabetes mellitus (T2DM) rodent model to investigate the protective effects of Pue against diabetic vasculopathy. Our research uncovered a novel role of Pue in mitigating LPS‐ATP or HG‐primed damage and pyroptosis in HUVECs. Pue effectively alleviated pyroptosis induced by LPS‐ATP by blocking ROS‐NLRP3 signalling. These findings suggested the clinical potential of Pue as a treatment option for diabetic vasculopathy.

## 2 MATERIALS AND METHODS

### 2.1 Materials

Puerarin (purity ≥98%), streptozotocin (STZ, purity ≥98%) and CoCI_2_ were obtained from Sigma Aldrich (MO, USA). CCK8 assay kit, LDH assay kit, ELISA Kits and Caspase‐1 activity assay kit was purchased from Beyotime (China). Annexin V Alexa Fluor 488‐PI was obtained from ThermoFisher (MA, USA). DCFDA probes were purchased from Yesen (Shanghai, China). Polyclonal antibodies against eNOS, p‐eNOS, NLRP3, GSDMA, Cleaved Caspase‐1, Caspase‐1, IL‐1β, IL‐18, Bax, Bcl‐2, CD31 and GAPDH were purchased from Abcam (MA, USA). PrimeScript® RT reagent Kit and SYBR® Premix Ex Taq™ were purchased from ACCURATE BIOLOGY (China).

### 2.2 Cell culture

HUVECs were obtained from the cell bank of the Chinese Academy of Science (Shanghai, China). They were grown to 70%–80% in endothelial growth medium (ECM) with 10% FBS and 1% penicillin–streptomycin antibiotics in an incubator before experimentation.

### 2.3 CCK‐8 assay

Various concentrations of Pue were added after cell adhesion. After 24 h, an agent from Cell Counting Kit‐8 (CCK8) (REF: C0037, Beyotime, China) was added and incubated for 2 h. The absorbance of each well at 450 nm is assessed.

### 2.4 Lactate dehydrogenase (LDH) activity assay

LDH assay kit (REF: C0016, Beyotime, China) was used to detect LDH activity referring to its instruction. The absorbance of each well at 450 nm is detected.

### 2.5 Caspase‐1 activity

After treatment, caspase‐1 activity was analysed in HUVECs using caspase‐1 activity assay kit referring to the supplier's instructions (REF: C1102, Beyotime, China).

### 2.6 Flow cytometry

Treated HUVECs were harvested and stained using 5 μL propidium iodide (PI) and 10 μL FITC. The results were analysed using FlowJo software.

### 2.7 Flow cytometric ROS assay

HUVECs were collected and washed twice with PBS, then incubated with DCFH‐DA (REF: 50101‐A, Yesen, Shanghai, China) for 30 min at 37°C in the darkness. After three washes in PBS, the cells were resuspended in 500 μL PBS, and measured using flow cytometer.

### 2.8 Animal experiments

All animal care was approved by the Animal Ethics Committee of the Shanghai University of Traditional Chinese Medicine (PZSHUTCM2303060002). SD male rats were purchased from Shanghai University of Traditional Chinese Medicine. All rats were housed in the SPF animal room of Shanghai University of Traditional Chinese Medicine. Except for the control group, each rat was received high‐fat diet (HFD) feeding for 4 weeks. After 12 h fasting, the rats were intraperitoneally injected with 100 mg/kg STZ. A week after STZ administration, rats with fasting blood glucose (FBG) concentration more than 16.7 mM were chosen for the following experiments.

Rats were randomized into the control group, T2DM group, Pue‐low dose group (100 mg/kg) and Pue‐high dose (150 mg/kg) with eight rats in each group. Pue dissolved in 0.5% CMC‐Na was delivered by oral gavage daily. After 21 days, animals were killed under anaesthesia with an intraperitoneal injection of 3% pentobarbital sodium. Then, the aorta tissue was collected for further experiments.

### 2.9 Quantitative Real‐Time reverse transcription PCR (RT‐PCT)

Total mRNA was extracted from HUVECs and the rat's aorta by TRIzol reagent and was transcribed into cDNA using cDNA Synthesis superMix. The cDNA was amplified using SYBR® Premix Ex Taq™ Kit by ABI PRISM® 7000 Real‐Time PCR System.

**Table:** 

Genes		
NLRP3	Forward	CCACAAGATCGTGAGAAAACCC
	Reverse	CGGTCCTATGTGCTCGTCA
CASPASE‐1	Forward	TTTCCGCAAGGTTCGATTTTCA
	Reverse	GGCATCTGCGCTCTACCATC
IL‐1β	Forward	TTCGACACATGGGATAACGAGG
	Reverse	TTCGACACATGGGATAACGAGG
IL‐18	Forward	TCTTCATTGACCAAGGAAATCGG
	Reverse	TCCGGGGTGCATTATCTCTAC

### 2.10 Western blot

The cells and tissues were lysed with RIPA buffer. Thirty microgram total protein was isolated with 10%–12.5% SDS‐polyacrylamide gel electrophoresis (SDS‐PAGE) and transferred onto polyvinylidene Fluoride (PVDF) membranes. Five per cent skim milk was then used to block the membranes for 1 h at room temperature. The following primary antibodies were used in this experiment: NLRP3 (1:1000), GSDMD (1:1000), Cleaved Caspase‐1 (1:1000), Caspase‐1 (1:1000), IL‐1β (1:1000), IL‐18 (1:1000), VCAM‐1 (1:1000) and GAPDH (1:5000). Subsequently, membranes were washed three times with TBST, for a total of 50 min and incubated with 1:5000 diluted HRP‐conjugated secondary antibody for 1 h at room temperature. Then, the membranes were washed three times with TBST for a total of 30 min. The protein bands were detected using enhanced electrochemiluminescence (ECL) reagents by using a digital luminescence image analyser. Quantitative analysis was performed with the software provided by fusion imaging system.

### 2.11 Enzyme‐linked immunosorbent assay (ELISA)

The levels of IL‐1β and IL‐18 in cell supernatant and aortic tissue were assessed using ELISA kits (IL‐1β REF: PI301, IL‐18 REF: PI553, Beyotime, China). Standard, blank and sample wells were prepared accordingly. Sample wells received 40 μL of sample diluent followed by 10 μL of the test sample, then incubated at 37°C for 30 min. Colour development was initiated by adding 50 μL of enzyme reagent, with absorbance readings at 450 nm obtained after zeroing with blank wells.

### 2.12 Lower expression and over expression of NLRP3 in HUVECs

The small‐interfering RNA (siRNA) oligonucleotides selective for NLRP3 was purchased from GeneChem. HUVECs were transfected with NLRP3 siRNA or the negative control siRNAs with Lipofectamine 2000. The sequence is as follows: NLRP3 (5′–3′) CAACAGGAGAGACCUUUAUTT, (3′–5′) AUAAAGGUCUCUCCUGUUGTT. The NLRP3 gene cDNA was inserted into the pcDNA3.1 plasmid vector through a cloning process.

### 2.13 Histology and immunofluorescence

The aortic tissues were fixed with 4% paraformaldehyde solution, then cut into sections using a Stadie‐Riggs tissue slicer. Haematoxylin and eosin (H&E) staining was employed to show atherosclerotic lesions. Precipoint M8 (Germany) software was applied to calculate intimal thickness. Additionally, immunostaining was employed on the aortic tissue for the expressions of Bax, Bcl‐2 and NLRP3. Samples were observed under the microscope.

### 2.14 Statistical analysis

The SPSS software was performed to statistical analysis. The data are presented as the mean ± SD. One‐way ANOVA followed by Tukey's post hoc test was performed to measure differences. *p* < 0.05 was recognized as statistically significant.

## 3 RESULT

### 3.1 PUE attenuated LPS‐ATP or HG‐primed HUVECs injury

**FIGURE 1:**
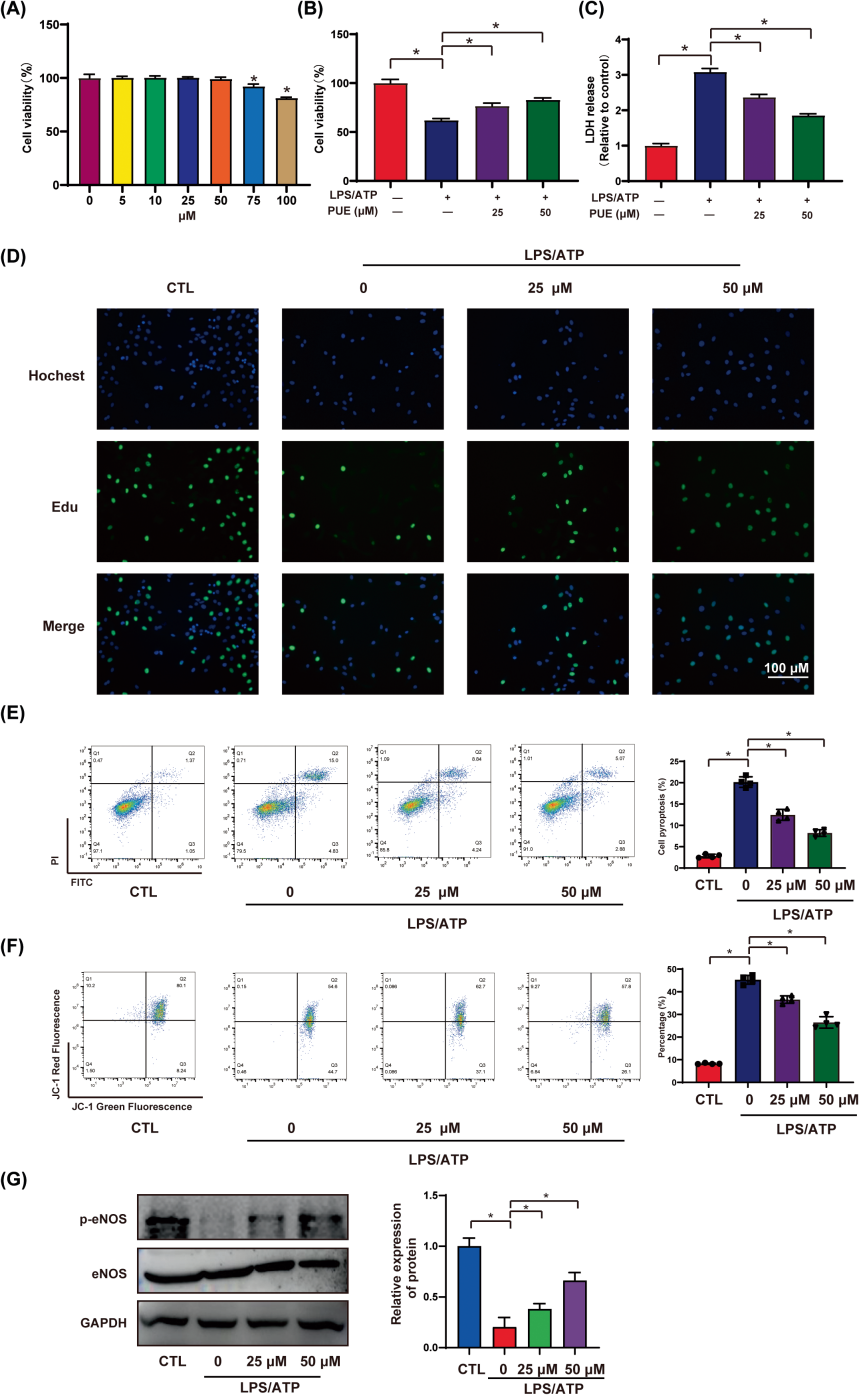
Pue attenuated LPS‐ATP‐primed HUVECs apoptosis HUVECs were pre‐treated with varying concentration of Pue for 2 h, then primed with 500 ng/mL LPS for 24 h and then stimulated with 5 mM ATP for 30 min. (A, B) The cell viability were detected using CCK8 assay kit. (C) The LDH release was measured using LDH assay kit. (D) The cell growth was examined by EdU assay kit. (E) Annexin FITC‐PI assay was performed to measure cell apoptosis. (F) Mitochondrial membrane potential was examined using JC‐1 assay kit. (G) The ratio of p‐eNOS‐eNOS were detected using western blot. Data are expressed as means ± SD. *p < 0.05.

Firstly, we investigated the influences of different concentrations of PUE on the viability of HUVECs. Based on the results of the CCK8 assay, 25 and 50 μM Pue was chosen for the most optimal concentration and used in subsequent experiments (Figure 1A). Previous studies showed that the anti‐inflammatory properties may contribute to the described cardiovascular benefits of procyanidins.^9^, ^10^, ^11^, ^12^ However, the molecular mechanisms underlying the anti‐inflammatory effects of PCB are not fully understood. To investigate the role of Pue in LPS‐ATP‐primed endothelial cells impairment, HUVECs were treated with varying concentration of Pue for 2 h and then exposed to LPS‐ATP for 24 h. LDH, an intracellular enzyme, releases into the cell supernatant upon cell damage. In this study, CCK8, LDH, Edu, Annexin FITC‐PI and JC‐1 assay kits were performed to measure cell death. As shown in Figure 1B–F, LPS‐ATP treatment remarkedly promoted HUVECs death, which was remarkedly reversed by Pue pretreatment.

Growing evidence indicates that eNOS, found in endothelial cells, plays a crucial role in synthesizing nitric oxide (NO), a key regulator of endothelium‐dependent relaxation. In our study, we assessed eNOS activity by examining the p‐eNOS (phosphorylated endothelial nitric oxide synthase) to eNOS ratio. The results demonstrated that in both models, the ratio of p‐eNOS‐eNOS decreased significantly in the presence of LPS‐ATP or HG. However, treatment with Pue partially reversed this decrease in the ratio of p‐eNOS‐eNOS (Figure 1H). The above results suggested that Pue has a protective effect on LPS‐ATP‐primed HUVECs impairment.

### 3.2 Pue pretreatment alleviated pyroptosis in LPS‐ATP and HG‐primed HUVCEs

**FIGURE 2:**
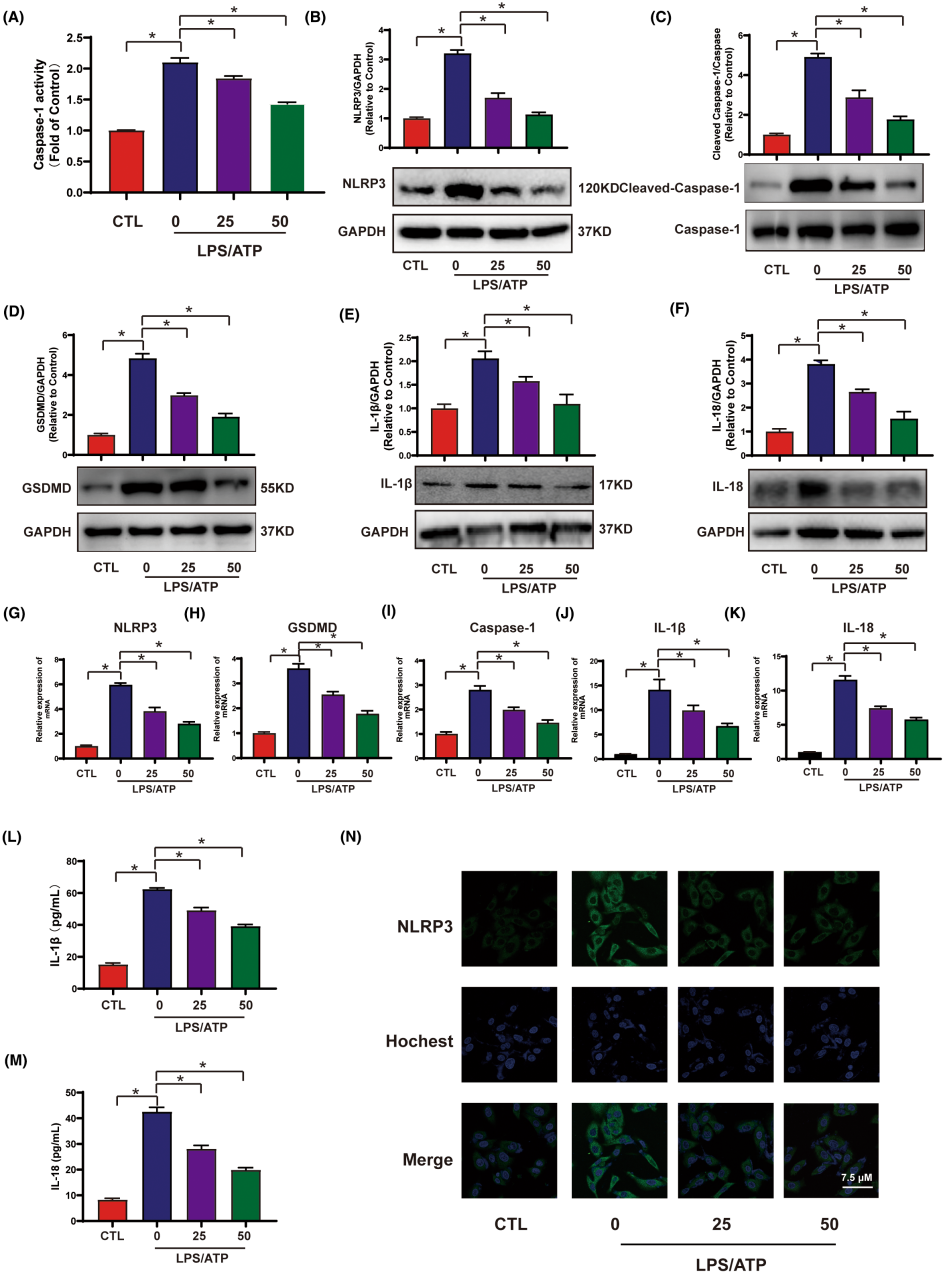
Pue repressed LPS‐ATP‐primed HUVECs pyroptosis. HUVECs were pre‐treated with varying concentrations of Pue for 2 h, then primed with 500 ng/mL LPS for 24 h and then stimulated with 5 mM ATP for 30 min (A) The activity of Caspase‐1 was tested using Caspase‐1 activity assay kit. (B–F) Western blot were performed to detect the protein expression of NLRP3, GSDMD, Cleaved Caspase‐1, Caspase‐1, IL‐1β and IL‐18. (G–K) The mRNA expression of NLRP3, GSDMD, Cleaved Caspase‐1, IL‐1β and IL‐18 were measured using RT‐PCR. (L, M) The release of IL‐1β and IL‐18 in cell supernatant were examined using ELISA kits. (N) The expression of NLRP3 was examined using immunofluorescence. Scale = 7.5 μm. Data are expressed as means ± SD. *p < 0.05.

Pyroptosis is caspase‐1‐dependent inflammatory cell death.^29^ To further investigate the effect of Pue on pyroptosis, caspase‐1 activity assay kit was employed. As illustrated in Figure 2A, the LPS‐ATP exposure contributed to an observable increase in caspase‐1 activity, while Pue remarkedly reduced the LPS‐HG‐ATP‐induced increase in caspase‐1 activity. Exposure to LPS‐HG‐ATP remarkedly up‐regulated the protein and mRNA expression of NLRP3, which was consistent with the previous reports (Figure 2B–G). However, 25 and 50 μM Pue suppressed the expression level of NLRP3. In addition, the expression of NLRP3 which was detected by confocal microscopy observed the same results. According to reports, NLRP3 is capable to induce the activation of GSDMD, IL‐1β and IL‐18 via cleavage of caspase‐1, thus we detected the protein and mRNA of cleaved‐caspase‐1‐caspase‐1, GSDMD, IL‐1β and IL‐18. We found that the presence of Pue inhibited the LPS‐HG‐ATP‐induced elevation of protein expression of Cleaved Caspase‐1, GSDMD, IL‐1β and IL‐18 by 0.75, 0.62, 0.57 and 0.67 folds respectively (Figure 2C–F). Consistently, the mRNA levels also were weakened after Pue pretreatment (Figure 2H–K). We subsequently used ELISA kits to detect the concentration of IL‐1β and IL‐18 released in cell supernatant induced by LPS‐ATP in HUVECs. The results clearly showed that the upregulated secretion of IL‐1β and IL‐18 decreased after Pue pretreatment (Figure 2I,M). Consistently, immunofluorescence results demonstrated that Pue pretreatment decreased the LPS‐ATP‐primed expression levels of NLRP3 (Figure 2N). Additionally, to confirm the effect of Pue in inhibiting pyroptosis, we also performed HG‐induced HUVECs model. Interestingly, Pue also mitigated HG‐induced HUVEC cells damage and pyroptosis (Figures S1 and S2). These findings support that Pue reduces LPS‐ATP and HG‐primed pyroptosis in HUVECs.

### 3.3 Pue pretreatment inhibited pyroptosis depending on ROS in LPS‐ATP and HG‐primed HUVCEs

**FIGURE 3:**
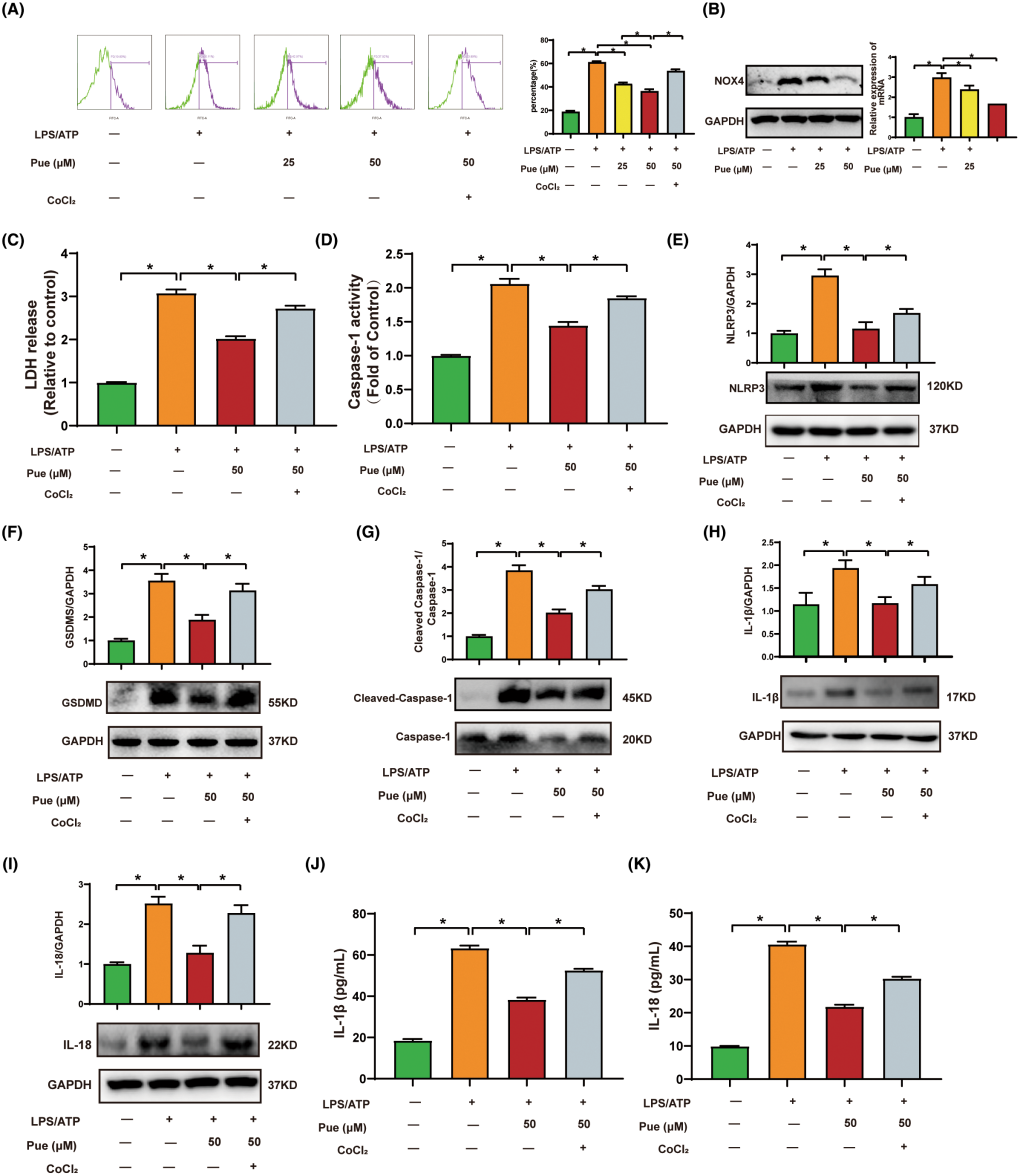
Pue repressed NLRP3 inflammasome activation and pyroptosis by repressing ROS generation. HUVECs were pre‐treated with varying concentrations of Pue or 500 CoCI_2_ for 2 h before primed with HUVECs (A) DCFH‐DA probe was subjected to examine the production of ROS. (B) The expression of NOX4 was detected using western blot. (C, D) The release of LDH and Caspase‐1 activity were measured by detection kits. (E–I) The levels of pyroptosis‐associated protein containing NLRP3, Cleaved Caspase‐1‐Caspase‐1, GSDMD, IL‐1β, and IL‐18 were determined by western blot. (J, K) The release of IL‐1β and IL‐18 in cell supernatant were determined by ELISA kit. Data are expressed as means ± SD. *p < 0.05.

ROS production is important for pyroptosis, which mediates the activation of NLRP3 inflammasome.^30^ Mounting evidence showed that HUVECs generate excessive amounts of ROS in the presence of LPS‐ATP that contributed in oxidative stress. Hence, we interrogated the influence of Pue on LPS‐ATP‐primed ROS generation. As presented in Figure 3A, the exposure of HUVECs cells to LPS‐ATP resulted in increase of intracellular ROS level, and a markable decrease of intracellular ROS. While Pue pretreatment significantly inhibited LPS‐ATP‐primed ROS production, the ROS inducer CoCI_2_ remarkedly enhanced the ROS generation. NADPH oxidase 4 (NOX‐4) plays a crucial role in generating ROS in the response to extracellular toxic damage. Here, we observed a significant up‐regulation in the expression of NOX4 at protein level upon exposure to LPS‐ATP. Treatment with Pue effectively decreased NOX‐4 expression (Figure 3B). Furthermore, Pue observably inhibited LPS‐ATP‐induced increase in LDH release and the activation of caspase‐1, which were reversed by CoCI_2_ treatment (Figure 3C,D). Furthermore, the protein expression level of pyroptosis‐associated marker in the LPS‐HG‐ATP treatment were dramatically enhanced compared with the control group. Whereas Pue pretreatment significantly inhibited LPS‐ATP‐ primed pyroptosis, and the ROS inducer CoCI_2_ markedly promoted the levels of pyroptosis‐associated marker (Figure 3E–I). Simultaneously, the release of IL‐1β and IL‐18 distinctively elevated in LPS‐ATP‐primed HUVECs, while Pue pretreatment could effectively reduce the production of IL‐1β and IL‐18 (Figure 3J,K). These findings indicate that ROS may mediate the inhibitory effect of Pue on NLRP3 inflammasome‐mediated pyroptosis in LPS‐ATP‐primed HUVECs.

### 3.4 Pue repressed NLRP3‐inflammation‐dependent pyroptosis

**FIGURE 4:**
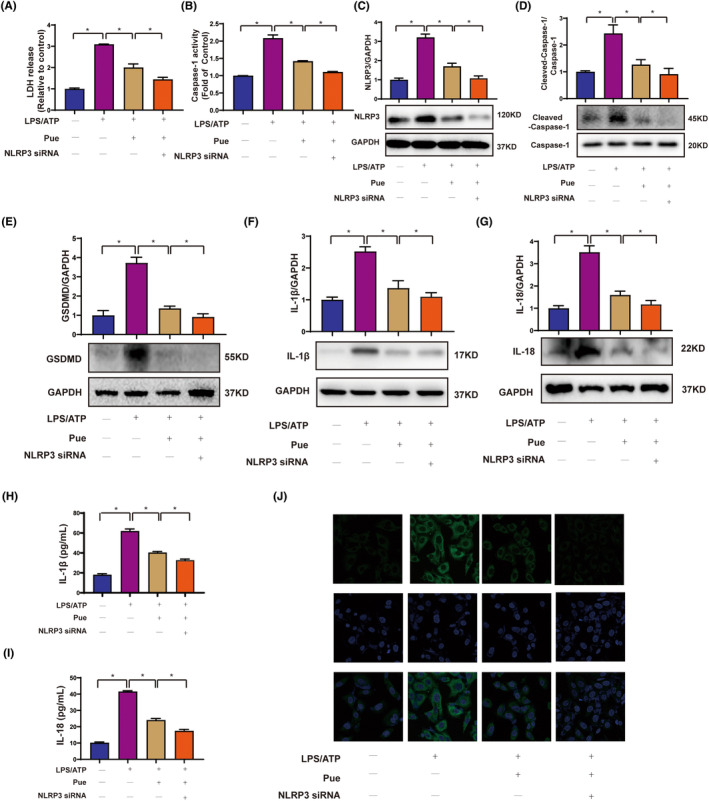
Pue alleviated pyroptosis dependent on NLRP3 inflammasome. HUVECs were transfected with NLRP3 siRNA for 24 h, then exposed to Pue for 2 h and following primed with 500 ng/mL LPS for 24 h and eventually stimulated with 5 mM ATP for 30 min. (A, B) The release of LDH and Caspase‐1 activity were measured by detection kits. (C–G) The levels of pyroptosis‐associated proteins containing NLRP3, Cleaved Caspase‐1‐Caspase‐1, GSDMD, IL‐1β and IL‐18 were determined by western blot. (H, I) The release of IL‐1β and IL‐18 in cell supernatant were determined by ELISA kits. (J) The expression of NLRP3 was examined using immunofluorescence. Scale = 7.5 μm. Data are expressed as means ± SD. *p < 0.05.

Aberrant NLRP3 inflammasome activation is closely related to pyroptosis.^29^ The above‐mentioned results indicated that NLRP3 inflammasome was repressed in LPS‐HG‐ATP‐primed HUVECs after Pue pretreatment. We further investigated the effects of NLRP3 on Pue function by silencing or overexpressing NLRP3. As presented in Figure 4A,B and Figure S2A,B, silencing of NLRP3 inflammasome remarkedly promoted Pue‐induce decrease in pyroptosis, while the effect was blocked after overexpressing NLRP3, as evidenced by the decreases in the release of LDH and Caspase‐1 (Figure 4A,B). Next, western blot and ELISA were employed to measure a series of pyroptosis‐related proteins containing NLRP3, GSDMD, Cleaved‐Caspase‐1‐Caspase‐1, IL‐1β and IL‐18. NLRP3 siRNA enhanced the inhibitory impact of Pue on the pyroptosis‐related protein level to LPS‐ATP‐primed HUVECs, while this effect was blocked after overexpressing NLRP3 (Figure 4C–I and Figure S2C,D). Likewise, confocal microscopy analysis confirmed that compared with LPS‐ATP‐primed group, Pue triggered a decrease in NLPR3 expression and NLRP3 siRNA combined with Pue further caused the downregulation of NLPR3 expression (Figure 4J), In contrast, the observed effect was reversed upon overexpression of NLRP3 (Figure S2E). Taken together, our results highlighted the important contribution of NLRP3 to Pue inhibition of pyroptosis.

### 3.5 Pue pretreatment protected against damage in aorta of T2DM

**FIGURE 5:**
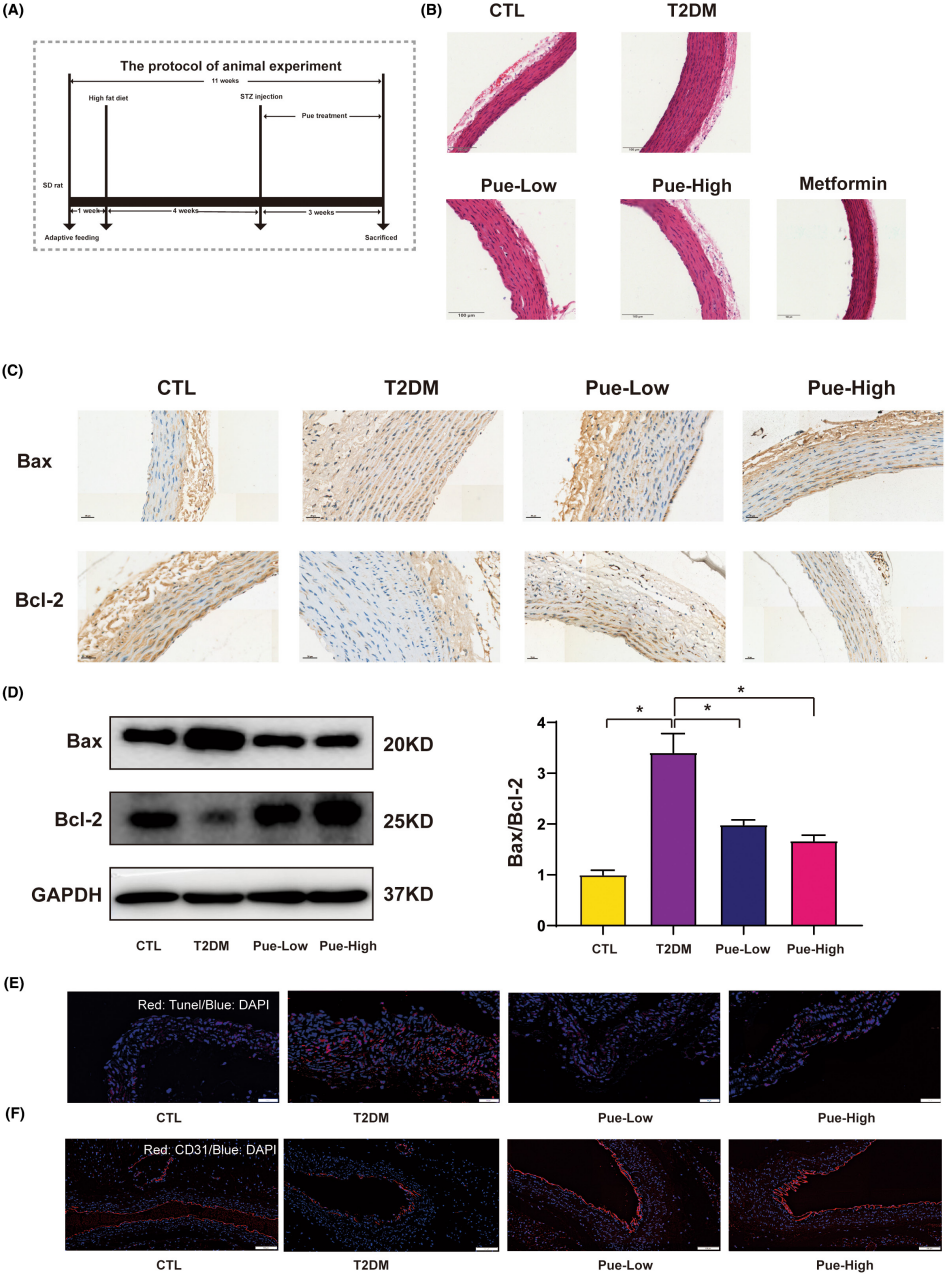
Pue treatment attenuated apoptosis in the aorta of T2DM rats T2DM rats were administrated with Pue (100 mg/kg/day or 150 mg/kg/day, i.g.) for 4 weeks (A) Schematic of the study design depicting 6‐week‐old male rats. (B) H&E staining was performed to detect aorta wall (Scale bar = 100 μm) (C, D) Immunohistochemistry staining and western blot were subjected to measure the expression of Bax‐Bcl‐2 in aorta of T2DM rats. (E) The samples were sectioned and processed using a TUNEL staining kit. (Scale bar = 50 μm) (F) Immunofluorescence staining was employed to detect the expression of CD31 (Scale bar = 100 μm). Data are expressed as means ± SD. *p < 0.05.

To dissect the role of Pue on diabetic‐induced aortas endothelial cell damage, STZ was injected into HFD‐fed male rats to induce T2MD. Schematic of the animal study design is illustrated in Figure 5A. The pathological changes of arterial vessels were observed by HE staining. Compared with the control group, the vascular wall in T2DM rat was significantly thicker. Pue administration decreased the thickness of the vascular wall (Figure 5B). The impacts of Pue were observed in the aorta of T2DM through the detection of apoptosis marker Bcl‐2 and Bax. Immunohistochemistry and western blot showed that T2DM rats presented obviously increased expression of Bax as well as obviously decreased expression Bcl‐2 in aorta, while Pue treatment obviously decreased Bax expression and increased Bcl‐2 expression in the aorta of T2DM rats (Figure 5C,D). As illustrated in Figure 5A, the T2DM group exhibited a significantly higher number of TUNEL‐positive nuclei in the aortic sections compared to the control group. However, treatment with Pue obviously reduced the number of TUNEL‐positive nuclei compared to the T2DM group. These findings indicate that Pue mitigated apoptosis of cells in the aorta of individuals with T2DM (Figure 5E). Subsequently, we detected the expression of CD31, a vascular endothelial‐specific marker, by immunofluorescence in aorta tissue. As illustrated in Figure 5F, the expression of CD31 was obviously increased in Pue treatment compared with the T2DM group. We concluded that Pue could mitigated apoptosis of cells in the aorta injury cell in T2DM rats.

### 3.6 Pue treatment alleviated pyroptosis in T2MD rats

**FIGURE 6:**
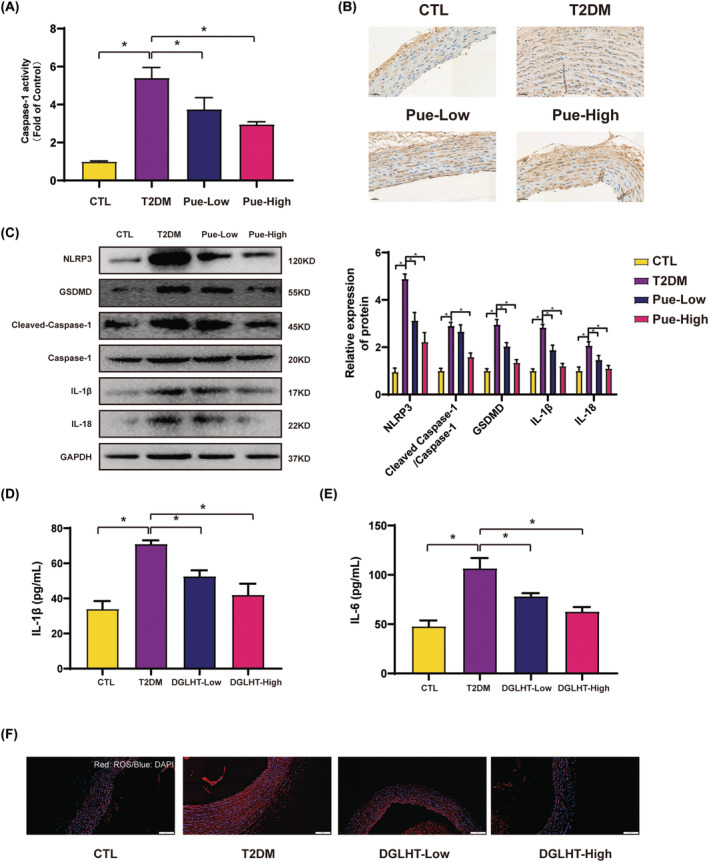
Pue suppressed pyroptosis in the aorta of T2DM rats T2DM rats were administrated with Pue (100 mg/kg/day or 150 mg/kg/day, i.g.) for 4 weeks (A) The activity of Caspase‐1 was measured by Caspase‐1 activity assay kit. (B) Immunohistochemistry staining were used to measure the expression level of NLRP3 in aorta wall. (C) Western blot was performed to detect the expression level of NLRP3, GSDMD, Cleaved Caspase‐1, Caspase‐1, IL‐1β and IL‐18 (D) The release of IL‐1β and IL‐18 in the serum samples were determined by ELISA kit. (E) Frozen aortic sections were incubated with 5 μmol/L DHE for 20 min and analysed using fluorescence microscopy (Scale bar = 100 μm). Data are expressed as means ± SEM. *p < 0.05.

For a comprehensive understanding of the protective effects of Pue in HUVECs, we further conducted in vivo experiments to confirm the mechanism. As expected, caspase‐1 activity in the T2DM group obviously increased compared to the control group, while Pue pretreatment alleviated caspase‐1 activity (Figure 6A). Furthermore, immunohistochemistry results revealed increased NLRP3 levels in T2DM rats, while Pue treatment mitigated these effects (Figure 6B,C). Western blotting results showed that, in T2MD rats, Pue treatment led to a remarkable decrease in the protein expression of NLRP3, Cleaved Caspase‐1, IL‐1β and IL‐18, indicating the inactivation of pyroptosis. Interestingly, secretion of IL‐1β and IL‐18 in the serum samples were remarkedly increased in T2DM rats, whereas Pue remarkedly decreased the release of IL‐1β and IL‐18 (Figure 6D,E). We also assessed the levels of ROS in the aortas using DHE staining. These results revealed that Pue treatment exhibited significantly decreased ROS levels in the aortas compared to the T2DM rat. Thus, we concluded that Pue can inhibit T2DM‐induced aorta pyroptosis, which was consistent with our in vitro findings.

## 4 DISCUSSION

Inflammation, implicated in the pathological development of various cardiovascular diseases, leads to endothelial dysfunction in a prolonged inflammatory state—a specific event in diabetes‐mediated vascular complications.^31^ For instance, the pretreatment of HUVECs with lipopolysaccharide LPS derived from *Escherichia coli* or *Salmonella*, well‐established inducers of pro‐inflammatory models both in vitro and in vivo, triggers the secretion of a spectrum of pro‐inflammatory cytokines. Notably, TNF‐α, MCP‐1, IL‐6 and IL‐8 are among the cytokines released, potentially accelerating the development of atherogenic lesions on the inner walls of arteries. The intervention in inflammation is a key aspect of diabetes‐mediated vascular complications. Hence, an in vitro model of LPS‐induced HUVECs was employed in this study. Our study demonstrated that Pue treatment inhibited pyroptosis in LPS‐ATP‐primed HUVECs. Furthermore, we discovered that the inhibition of ROS and the NLRP3 inflammasome was crucial in the protective mechanism of Pue. These data validated that Pue exerts a protective effect on the initiation, progression and consequences of diabetes‐mediated vascular complications.

Accumulating evidence indicated that the exposure of LPS‐ATP to HUVECs caused endothelial cell apoptosis.^32^, ^33^ We firstly demonstrated that Pue exerts a protective effect on LPS‐ATP‐primed apoptosis in HUVECs. Pyroptosis is programmed cell death inflammation and has been validated important for promoting diabetic vasculopathy.^34^ Pyroptosis induced by the NLRP3 inflammasome is a pro‐inflammatory cell death mechanism characterized by recruiting and cleaving caspase‐1, and cleaved caspase‐1 can then processes GSDMD, IL‐1β and IL‐18 to their mature forms.^35^ Pyroptosis‐related proteins, GSDMs generate porous membrane channels, leading to cell membrane rupture and the subsequent release of inflammatory factors, resulting in more severe inflammatory responses. Long non‐coding RNAs (LncRNAs) are non‐coding RNA molecules with more than 200 nucleotides in eukaryotes. These non‐coding RNAs play diverse biological roles, including acting as molecular scaffolds in the nucleus, assisting in variable splicing, regulating chromosomal structure, controlling translation and either promoting or inhibiting messenger RNA (mRNA) degradation in the cytoplasm. Several reports showed that LPS‐ATP treatment could contribute to the occurrence of pyroptosis in HUVECs. Hence, to explore the effect of Pue in pyroptosis of HUVECs, LPS and ATP were used to mimic the inflammatory microenvironment of the aortic endothelium. We observed that Pue pretreatment significantly attenuated LPS‐ATP‐primed pyroptosis in HUVECs.

Overwhelming accumulation of ROS can impart cells and tissues, contributing to the progression of the diseases such as diabetes and bowel disease.^36^, ^37^ Hyperglycaemia and inflammation hampers insulin signalling and eNOS activity within endothelial cells. This impairment leads to the accumulation of ROS and triggers the synthesis of pro‐inflammatory factors through pathways involving polyols, hexosamines and advanced glycation end products (AGEs). Production of ROS is a dominant upstream regulator of NLRP3 inflammation activation.^38^, ^39^, ^40^ Pue has been reported to exert antioxidative stress in multiple diseases. In agreement with this view, this study indicated that Pue treatment reduced ROS production, which may be an upstream mechanism regulating the NLRP3 inflammasome and pyroptosis‐related protein, which can be repressed by CoCI_2_, a ROS inducer.

NLRP3 inflammasome has been implicated in various diabetic cardiovascular complications, and its activation has recently been indicated as a possible marker of cardiovascular risk.^41^ We found here that the expression of both NLRP3 and pyroptosis‐related protein was inhibited in LPS‐ATP‐primed HUVECs. In addition, we further explored that the changes in NLRP3 inflammasome affected the consequence of Pue in LPS‐ATP‐primed cell pyroptosis. Silencing or overexpressing of NLRP3 inflammasome enhanced or alleviated the inhibitory influence of Pue on LPS‐ATP‐primed pyroptosis, suggesting that NLRP3 inflammasome is required for Pue to repress LPS‐ATP‐primed pyroptosis.

Noteworthy, the mechanism of pyroptosis involving Pue treatment for diabetic vasculopathy was explored. Firstly, we found that Pue obviously promoted the vascular wall intimal thickness of blood vessel. Anti‐apoptosis effect of Pue was observed in the aorta of T2DM rats, as evidenced by the inhibition of Bax, Tunel and promotion of Bcl‐2. Endothelial cells are a specialized type of cells with distinctive morphological, functional and genetic features. During this process, the cells undergo significant morphological and functional changes, losing their endothelial marker CD31. CD31 was obviously decreased in the aortic section of T2DM rat compared to the control group. After treatment with Pue, the expression levels of CD31 were restored. Pue was able to inhibit pyroptosis‐associated marker level in the aortas tissue and reduced secretion of IL‐1β and IL‐18 in the serum samples which at least partially support the hypothesis that Pue can suppress T2DM‐induced vascular complications by inhibiting pyroptosis. Notably, consistent with the in vitro results, we observed that Pue also exhibited the ability to inhibit ROS generation in the aorta T2DM rats in vivo.

To summarize, Pue exerted a protective effect on HUVECs and diabetic vasculopathy by inhibiting pyroptosis, which was mediated by modulation of the ROS‐NLRP3 signalling pathways. This study provided new evidence for Pue‐based treatment of vascular complications of diabetes.

## AUTHOR CONTRIBUTIONS

**Huizhen Wei:** Data curation (equal); formal analysis (equal); investigation (equal); writing – original draft (equal). **Mengru Sun:** Data curation (equal); formal analysis (equal); investigation (equal); writing – original draft (equal). **Ruixuan Wang:** Data curation (equal). **Hairong Zeng:** Funding acquisition (equal); project administration (equal); resources (equal). **Bei Zhao:** Funding acquisition (equal); project administration (equal); resources (equal); writing – review and editing (equal). **Shenyi jin:** Funding acquisition (equal); Project administration (equal); Resources (equal); Writing‐review & editing (equal).

## FUNDING INFORMATION

This study was funded by Shanghai Sailing Program, Grant‐Award Number: 23YF1442700; National Natural Science Foundation of China, Grant‐Award Number: 82305097.

## CONFLICT OF INTEREST STATEMENT

The authors have declared that no competing interest exists.

## Supporting information


Figure S1 and S2.

## Data Availability

The data that support the findings of this study are available from the corresponding author upon reasonable request.
