# Supplementary material for: Puerarin mitigated LPS‐ATP or HG‐primed endothelial cells damage and diabetes‐associated cardiovascular disease via ROS‐NLRP3 signalling
Source: J Cell Mol Med. 2024 May 22;28(10):e18239. doi: 10.1111/jcmm.18239 (PMC11109626; doi:10.1111/jcmm.18239)
Supplement: Supplementary file 1 — Figure S1 and S2. [file JCMM-28-e18239-s001.docx]

**Supplementary materials**

**Puerarin mitigated LPS-ATP or HG-primed endothelial cells damage and diabetes-associated cardiovascular disease via ROS-NLRP3 signaling**

**Huizhen Wei^1^** **| Mengru Sun^2^** **| Ruixuan Wang^2^** **| Hairong Zeng^2,*^** **| Bei Zhao^2,*^** **|Wenjun Sha^1,*^**

^1^Department of Endocrinology and Metabolism, Putuo Hospital, Shanghai University

of Traditional Chinese Medicine, Shanghai, China

^2^Shanghai Frontiers Science Center of TCM Chemical Biology, Institute of Interdisciplinary Integrative Medicine Research, Shanghai University of Traditional Chinese Medicine, Shanghai, China

**Correspondence**

Zenghai Rong, Shanghai Frontiers Science Center of TCM Chemical Biology, Institute of Interdisciplinary Integrative Medicine Research, Shanghai University of Traditional Chinese Medicine, Shanghai, China

E-mail: [hr2323631@126.com](mailto:hr2323631@126.com)

Bei Zhao, Shanghai Frontiers Science Center of TCM Chemical Biology, Institute of Interdisciplinary Integrative Medicine Research, Shanghai University of Traditional Chinese Medicine, Shanghai, China

E-mail: [zhaobei0207@shutcm.edu.cn](mailto:zhaobei0207@shutcm.edu.cn)

Wenjun Sha, Department of Endocrinology and Metabolism, Putuo Hospital, Shanghai University of Traditional Chinese Medicine, Shanghai, China

E-mail: 13916150913@126.com

**Funding information**

Shanghai Sailing Program, Grant/Award Number: 3YF1442700; National Natural Science Foundation of China, Grant/Award Number: 81704270; Shanghai Municipal Health Commission, Grant/Award Number: 20204Y0154; Training Program for High-caliber Talents of Clinical Research at Affiliated Hospitals of SHUTCM Grant/Award Number: 2023LCRC20; Training Program for for Basic Research at Putuo Hospital of SHUTCM, Grant/Award Number: 2022-RCQH-02.

**Abbreviations**

Pue, puerarin; LPS, lipopolysaccharide; ATP, adenosine triphosphate; NLRP3, NLR family pyrin domain containing 3; ROS, reactive oxygen species; T2DM, type 2 diabetes mellitus


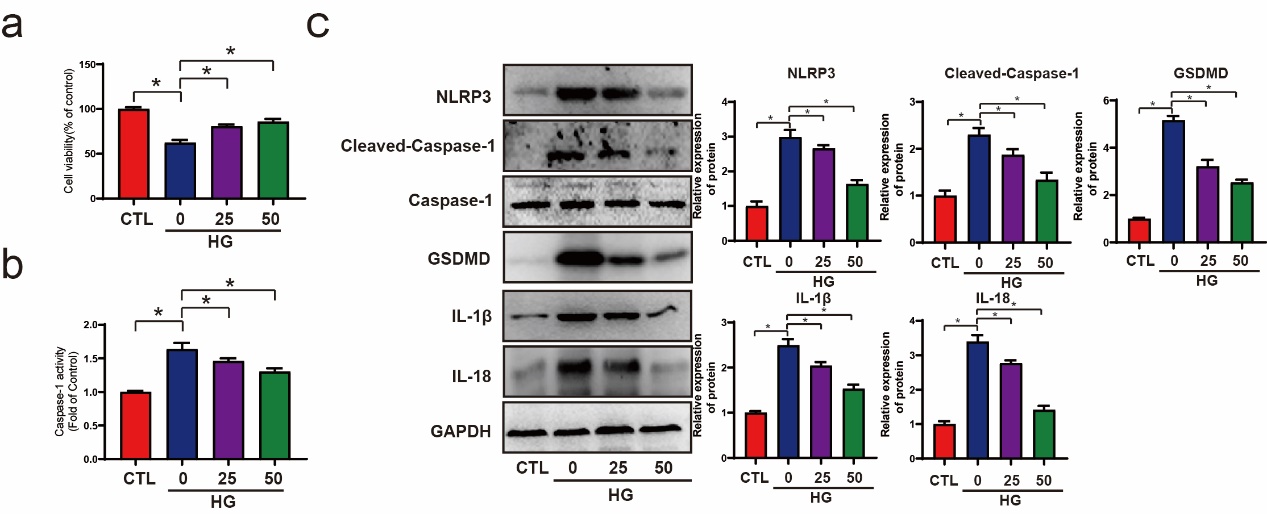
Figure S1

Figure S1 Pue pretreatment inhibited pyroptosis in HG-primed HUVCEs HUVECs were pre-treated with varying concentrations of pue for 2 h, then primed with 50 mM HG for 24 h. (a) The cell viability were detected using CCK8 assay kit. (b) The activity of Caspase-1 was tested using Caspase-1 activity assay kit. (c) Western blot were performed to detect the protein expression of NLRP3, GSDMD, Cleaved Caspase-1, Caspase-1, IL-1β and IL-18. Data were expressed as means ± SD. **P*< 0.05


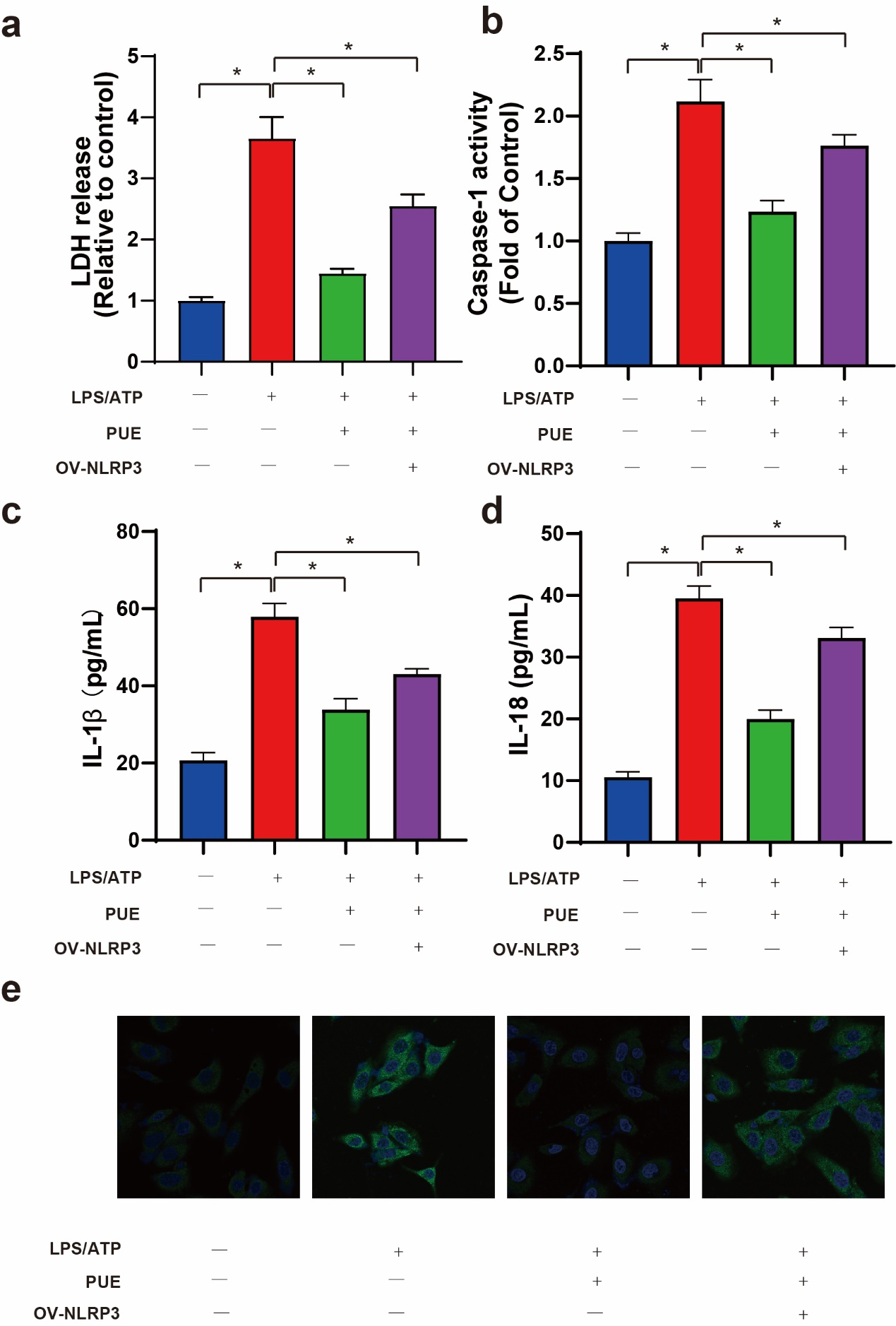
Figure S2

Figure S2 Pue alleviated pyroptosis dependent on NLRP3 inflammasome. HUVECs were transfected with NLRP3-overexpressing plasmid for 48 h, then exposed to pue for 2 h and following primed with 500 ng/ml LPS for 24 h and eventually stimulated with 5 mM ATP for 30 min. (a, b) The release of LDH and Caspase-1 activity were measured by detection kits. (c, d) The content of IL-1β and IL-18 were detected using Elisa kits. (e) The expression of NLRP3 was examined using immunofluorescence. Scale=7.5 μm. Data were expressed as means ± SD. **P*< 0.05
